# Single-Joint Type Hybrid Assistive Limb for Knee Training in the Acute Postoperative Phase After Opening Wedge High Tibial Osteotomy: A Feasibility and Safety Trial

**DOI:** 10.7759/cureus.60122

**Published:** 2024-05-11

**Authors:** Yuichiro Soma, Tomokazu Yoshioka, Shigeki Kubota, Hisashi Sugaya, Yukiyo Shimizu, Yasushi Hada, Masashi Yamazaki

**Affiliations:** 1 Physical Medicine and Rehabilitation, Institute of Medicine, University of Tsukuba, Tsukuba, JPN; 2 Orthopaedic Surgery, Institute of Medicine, University of Tsukuba, Tsukuba, JPN; 3 Physical Medicine and Rehabilitation, and Clinical Neurophysiology, Institute of Medicine, University of Tsukuba, Tsukuba, JPN

**Keywords:** feasibility and safety trial, visual analogue scale, the single-joint hybrid assistive limb, opening wedge high tibial osteotomy, knee extension angle

## Abstract

Background and objective: Opening wedge high tibial osteotomy (OWHTO) influences the knee extensor mechanism, the range of passive motion of knee extension and persistent quadriceps, and anterior knee pain and weakness. Rehabilitation should focus on quadriceps strength and improving joint mobility. The single-joint hybrid assistive limb device (HAL-SJ) is a wearable exoskeleton cyborg. In this study, we investigated the feasibility and safety of HAL-SJ training after the early postoperative period following OWHTO and whether the use of this device can improve functional outcomes, including knee muscle extensor strength and knee extension range of motion without knee pain.

Methods: Patients who had been diagnosed with knee osteoarthritis and had undergone OWHTO were assessed for eligibility in this prospective trial conducted at our institution between June 2015 and November 2020. The participants were split into two groups, i.e., 10 patients in the hybrid assistive limb (HAL) group and eight patients in the control group. We initiated HAL-SJ therapy on postoperative day 8 and continued it until the patient's discharge. During the hospitalization period, patients engaged in HAL-SJ-assisted knee extension exercises. This exercise routine encompassed five sets, each comprising 10 repetitions, and was conducted twice a week. We conducted assessments aimed at detecting any potential adverse events that could be linked to HAL training. Assessment of the knee extension angle via the visual analog scale (VAS) and strength assessments using a hand-held dynamometer (HHD) were conducted. To compare clinical outcomes before and after OWHTO, knee extension angle, the VAS, HHD, Japanese Orthopaedics Association (JOA) score, and the Japanese Knee Osteoarthritis Measure (JKOM) were assessed at four distinct time points.

Results: No adverse events were observed during the study. The assessment of clinical outcomes before and after OWHTO demonstrated a gradual improvement in outcomes.

Conclusion: The single-joint hybrid assistive limb device in patients who underwent OWHTO appears to be potentially safe. It contributed to enhanced muscle activity efficiency by reducing knee pain and improving knee extension angles in the early postoperative phase.

## Introduction

Opening wedge high tibial osteotomy (OWHTO) is a surgical procedure whereby a tibial osteotomy is performed from the medial to the lateral side of the proximal tibial tuberosity [1]. It is a joint-sparing surgery that preserves physiological knee joint function. Osteotomy of the proximal tibial tuberosity results in its enlargement. Postoperative modifications in patellar alignment, encompassing parameters such as patellar shift, tilt, and displacement, in conjunction with alterations in patellar height and tracking, play a pivotal role in influencing kinematic adaptations within the patellofemoral joint. Consequently, these adjustments contribute to anterior knee pain attributed to heightened stresses and increased patellofemoral pressure [2-5]. The OWHTO surgery influences the knee extensor mechanism. A previous study demonstrated that the range of passive motion of knee extension reached baseline values 12 weeks after OWHTO [6].

Persistent quadriceps weakness, anterior knee pain, and impaired knee kinematics after realignment suggest that the movement strategy may perpetuate joint destruction and impede the long-term success of realignment. Rehabilitation should focus on quadriceps strength and improved joint mobility to recover long-term function in individuals with medial knee OA. Although there are reports detailing rehabilitation programs customized to the specific needs of particular surgical procedures [7-9], there is an absence of reports regarding the existence of a dedicated rehabilitation regimen with a primary focus on targeting the knee extensor mechanism for individuals undergoing OWHTO.

The single-joint hybrid assistive limb device (HAL-SJ) is a wearable exoskeleton cyborg. It provides real-time assistance for knee joint movements by utilizing actuators mounted on the knee joints. Our previous case reports described knee joint extension training using HAL-SJ to provide rehabilitation treatment targeted at improving knee joint function after OWHTO or total knee arthroplasty (TKA) [10-11]. In previously described cases, we obtained an immediate improvement in the knee range of motion (ROM) without increasing knee pain. These case reports are novel as they document the progression from preoperative to postoperative recovery of knee extension ROM. Another study indicated that HAL-SJ training resulted in improved knee joint extensor movement during walking without inducing pain [12]. Nevertheless, it is important to acknowledge that these findings stem from case studies. Therefore, there exists a need to substantiate the impact of HAL-SJ training following surgical procedures through more controlled investigations.

We hypothesize that early intervention with HAL-SJ after knee surgery will reduce medical costs through the early recovery of knee function. Our hypothesis suggests that HAL-SJ training could represent an innovative rehabilitation intervention for patients in the early phase after OWHTO. This intervention is believed to have the potential to influence the knee extensor mechanism, thereby affecting the descent of the patella following OWHTO, ultimately resulting in a reduction of patellofemoral joint pressure and alleviation of anterior knee pain. To date, there have been no clinical trials assessing the utilization of HAL-SJ in patients who have undergone OWHTO. In this study, we aimed to evaluate the feasibility and safety of HAL-SJ training during the early postoperative phase following OWHTO and whether the use of this device can improve functional outcomes.

## Materials and methods

Enrollment of patients

**Figure 1:**
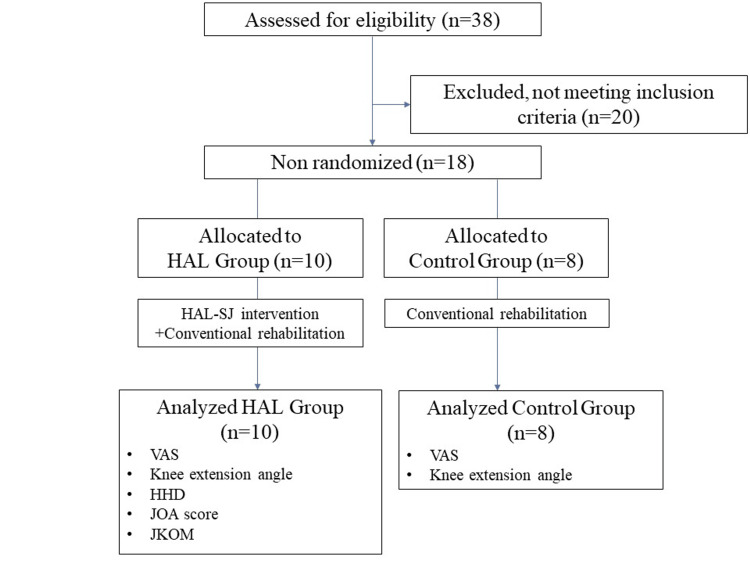
Flow chart depicting the therapeutic intervention and assessments for each patient group HAL: Hybrid assistive limb, HAL-SJ: Single-joint hybrid assistive limb device, VAS: Visual analog scale, HHD: Hand-held dynamometer, JOA: Japanese Orthopaedics Association, JKOM: Japanese Knee Osteoarthritis Measure

We conducted a prospective, non-randomized, controlled trial at our institution between June 2015 and November 2020. During this time, all patients who had been diagnosed with knee osteoarthritis and who underwent OWHTO were assessed for eligibility (Figure 1). Patients who underwent a primary OWHTO, who could understand an explanation of the study and provide informed consent, and who were available for observation throughout the study were included. Patients with severe deformities of the skeletal system other than in the surgical site, those for whom the wearing and training using the HAL was expected to be difficult because of underlying disease, and those with perioperative complications were excluded. Ten cases met the inclusion criteria of the HAL group prematurely, following which all high tibial osteotomy (HTO) participants were assigned to the control group. The HAL group consisted of 10 patients, and the control group consisted of eight patients (Table 1). These patients had undergone OWHTO using TomoFix Osteotomy System (DePuy Synthes, Raynham, MA, USA), artificial bone (OSferion60, a β-tricalcium phosphate from Olympus Terumo Biomaterials Corp., Tokyo, Japan), and biplanar osteotomy as described by Takeuchi et al. [13]. The actual enlarged angle and distance of osteotomy were 11.7^o^ and 12.3 mm in the HAL group, and 9.6^o^ and 11.7 mm in the control group. There were no significant differences between groups in demographic or surgical data (Table 1). 

**Table 1:** Patient descriptive data A p-value < 0.05 was considered significant. HAL: Hybrid assistive limb

Variables (values are mean ± SD)	HAL group (n = 10)	Control group (n = 8)	p-value
Age (years)	60.1 ± 7.5	55.6 ± 6.2	0.114
Male/female (n)	3/7	3/5	0.484
Height (cm)	161.7 ± 8.0	163.8 ± 9.2	0.206
Body weight (kg)	65.2 ± 12.3	77.4 ± 14.6	0.082
BMI (kg/m2)	24.8 ± 3.5	28.8 ± 4.0	0.081
Actual enlarged angle (degrees)	11.7 ± 2.7 (5.5 to 15.0)	9.6 ± 1.8 (8.0 to 13)	0.055
Distance of osteotomy (mm)	12.3 ± 2.5 (7.0 to 17.0)	11.7 ± 1.4 (11.0 to 15)	0.287

Therapeutic intervention

The HAL-SJ Treatment Program

We located the quadriceps muscles on the patient's thigh and placed electrodes on each muscle to capture the bioelectric potentials along the longitudinal axis of the muscle belly. Patients were then instructed to perform knee extensions and contract their quadriceps. Based on the recorded data, we guided the patients in simulating knee extension training, which they would undergo postoperatively. During the simulation, patients sat with their lower legs hanging naturally, adjusting the chair height so that their feet were not touching the floor. Patients performed 10 knee extensions with the assistance of HAL-SJ, and we identified the quadriceps muscle with the highest recorded bioelectric potential for further analysis [10-11].

The cybernic voluntary control (CVC) mode of HAL-SJ is designed to facilitate the patient's voluntary movements by integrating their voluntary muscle activity with the assistive torque provided to the knee joint. In our study, we implemented the CVC mode to allow the operator to fine-tune the level of physical support to ensure the patient's comfort while progressively reducing assistance as the training regimen advanced. The gain in assistive torque at each joint in response to the bioelectrical signals was controlled by a therapist so that the patient could move the knee joint sufficiently and easily within the ROM without aggravating pain and so the extension lag was as normal as possible [14]. In addition to the standard rehabilitation program, we initiated HAL-SJ intervention from postoperative day 8 and continued until the patient's discharge. The patients engaged in HAL-SJ-assisted knee extension exercises while in a seated position. This exercise routine encompassed five sets, each comprising 10 repetitions with 20 to 30-second breaks between sets, conducted twice a week. The HAL-SJ intervention was administered separately from the conventional rehabilitation intervention on the same day.

Conventional Rehabilitation

Conventional rehabilitation was initiated on the day after surgery and was overseen by a physical therapist. This rehabilitation regimen was administered five days a week, with each session lasting 40 minutes. The rehabilitation protocol encompassed a range of activities, including continuous passive motion exercises to enhance ROM, strength training for the hip and knee muscles, cryotherapy, and weight-bearing exercises. Weight-bearing exercises commenced on postoperative day 14. We did not control the rehabilitation program to increase the external validity of our findings.

Measurement

We conducted assessments aimed at detecting any potential adverse events that could be linked to HAL training. Adverse events, in this context, were defined as any unanticipated or unfavorable symptoms, illnesses, or indications of such conditions. This definition also encompassed abnormalities observed in clinical laboratory data. The monitored adverse events encompassed a range of critical outcomes, including mortality, life-threatening conditions, disabilities or incapacities, potential disabilities, and instances requiring hospitalization. Specific metrics were evaluated at each HAL-SJ session. These metrics included parameters such as knee extension angle, assessments via the visual analog scale (VAS), strength measured using a hand-held dynamometer (HHD), number of interventions administered, and the duration of HAL-SJ training throughout the hospitalization period. The HHD assessments were conducted before and after each HAL-SJ training session, providing immediate evaluations before and after intervention. Knee pain levels were quantified for both HAL and control groups using the VAS before and after intervention. The VAS assessments were carried out at the initiation of each HAL-SJ training and conventional rehabilitation sessions. Subsequently, after the HAL-SJ training or conventional rehabilitation sessions, the patients were queried about the intensity of knee pain.

Goniometry was employed to measure knee ROM, utilizing specific landmarks for accuracy. The landmarks included the greater trochanter of the femur, the proximal head of the fibula, and the lateral malleolus. Goniometry was chosen due to its reported reliability; it offers a precision of up to 1.0°, surpassing visual observation methods [15]. Knee extension angle assessments also were conducted before and following intervention for both groups. The maximum isometric knee extensor strengths in the surgical site leg were assessed using an HHD [16]. The HHD is a straightforward tool widely employed in clinical practice to objectively measure muscle strength. Maximum force was recorded in kilogram-force (kgf). Participants were instructed to be seated in an upright position with the knee positioned at a 90° flexion angle. The HHD was attached 10 cm proximal to the lateral malleolus and secured with an inelastic strap fastened around the therapy bed. The length of the strap allowed for anisometric contraction to be performed with the knee at 90° of flexion during testing. Participants were guided to extend their legs for 5 seconds, with strong verbal encouragement provided to ensure maximal effort. To determine the moment value, the lever arm, representing the length between the knee joint and the HHD, was manually measured and subsequently normalized to the participant's body mass (kgf/kg). The average value from the three measurements was used in the statistical analysis [17].

To compare clinical outcomes before and after OWHTO, various parameters including knee extension angle, the VAS score, HHD, JOA score [18], and the Japanese Knee Osteoarthritis Measure (JKOM) [19] were assessed in the HAL group at four distinct time points: before the surgery, on the day of discharge, and at one and three months postoperatively. The JOA scores evaluate subjective knee pain on a scale of 0 to 100 points in total, with a higher score indicating less pain. The score encompasses four ranges: pain on walking (0 to 30 points), pain on ascending or descending stairs (0 to 25 points), ROM (0 to 35 points), and joint effusion (0 to 10 points). Knee pain severity and self-reported physical function were assessed using the JKOM, a patient-based self-administered evaluation scoring system. The JKOM evaluates pain and stiffness (eight questions, 0 to 32 points), activities of daily living (ADL) (10 questions, 0 to 40 points), participation in social activities (five questions, 0 to 20 points), and general health conditions (two questions, 0 to 8 points). The maximum score in this person-specific assessment is 100 points [20].

Informed consent and ethical approval

The patients received a personalized explanation of the study, their participation requirements, and data usage before signing an informed consent form. Those included in this study agreed to participate and provided both oral and written informed consent. This study was approved by the Ethics Committee of the Tsukuba University, Faculty of Medicine (approval no.TCRB18-38). This study was conducted according to the principles of the World Medical Association (WMA) Declaration of Helsinki-Ethical Principles for Medical Research Involving Human Subjects with the amendments made in Seoul, South Korea, in October 2008, with a note of clarification on paragraph 29 added by the WMA General Assembly in Washington (2002) and a note of clarification on paragraph 30 added by the WMA General Assembly in Tokyo (2004). This study was also conducted per the Japanese Medical Research Involving Human Subjects Act and other guidelines, regulations, and acts. This study was registered with the University Hospital Medical Information Network (UMIN) clinical trials registry (UMIN000017012).

Statistical analysis

The independent samples t-test and Fisher’s exact test were utilized to compare the patients’ clinical characteristics between the HAL and control groups for each measurement. In the HAL group, the independent-samples t-test was utilized for the HHD assessments both before and after each HAL-SJ training session. A two-way mixed analysis of variance was employed to compare the differences in the VAS and knee extension angle (from before the surgery to the day of discharge) in the HAL and control groups. If the main effects of the repeated measures factor (time effect) or interactions were significant (p < 0.05), Tukey’s honestly significant difference was used for within-group comparisons of the outcomes at the various assessment time points. If the main effects of the between-subjects factor (intervention effect) or interactions were significant, the independent samples t-test was used for comparison between groups at the various assessment time points. To assess the relationship between the VAS score and the knee extension angle in the HAL and control groups, Pearson or Spearman correlation coefficients were employed to gauge the strength of a linear association. Additionally, in the HAL group, these correlations were used to assess the relationship between the VAS score and the HHD assessment or the knee extension angle and HHD assessment to gauge the strength of a linear association. The statistical analysis was performed using SPSS Statistics version 29 (IBM Corp., Armonk, NY, USA). The alpha level was set at p < 0.05.

## Results

No serious adverse events related to HAL training were observed in any session. There were no complications during the preparation, surgery, or rehabilitation process. The average hospitalization period was 23.2 ± 5.3 days in the HAL group and 21.8 ± 3.4 days in the control group. The mean number of HAL training sessions was 3.7 ± 1.1 times, with an average training duration of 15.3 ± 4.4 minutes. The comparison of clinical outcomes before and after OWHTO in the HAL group is presented in Table 2.

**Table 2:** Comparison of clinical outcomes before and after OWHTO in the HAL group OWHTO: Opening wedge high tibial osteotomy, HAL: Hybrid assistive limb, VAS: Visual analog scale, HHD: Hand-held dynamometer, JOA: Japanese Orthopaedics Association, JKOM: Japanese Knee Osteoarthritis Measure

Variables (values are mean ± SD)	Before surgery (range)	Day of discharge (range)	One month postoperative (range)	Three months postoperative (range)
VAS pain score	24.3 ± 24.4 (0 to 60)	5.5 ± 5.0 (0 to 18)	8.3 ± 11.2 (0 to 29)	7.8 ± 21.6 (0 to 69)
Extension angle (degrees)	–7.7 ± 6.2 (–15.0 to 0.0)	–4.4 ± 3.5 (–12.0 to 0.0)	–3.1 ± 3.7 (–11.0 to 0.0)	–1.2 ± 3.8 (–12.0 to 0.0)
HHD (kgf/kg)	0.30 ± 0.13 (0.14 to 0.54)	0.17 ± 0.07 (0.09 to 0.31)	0.24 ± 0.08 (0.15 to 0.35)	0.29 ± 0.10 (0.21 to 0.49)
JOA score	73.9 ± 8.2	54.0 ± 12.2	68.9 ± 10.8	78.0 ± 10.6
JKOM	31.9 ± 12.8	46.2 ± 18.6	38.2 ± 16.6	23.6 ± 13.2

**Figure 2:**
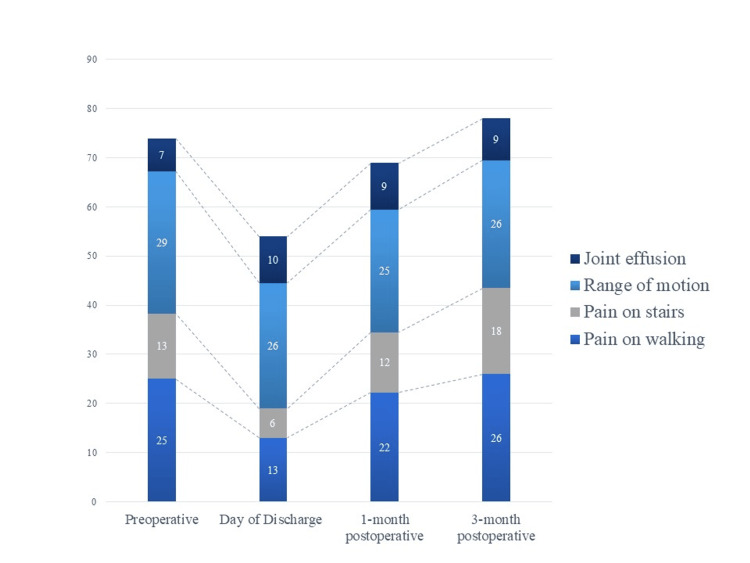
Breakdown of the results of the JOA score JOA: Japanese Orthopaedics Association

**Figure 3:**
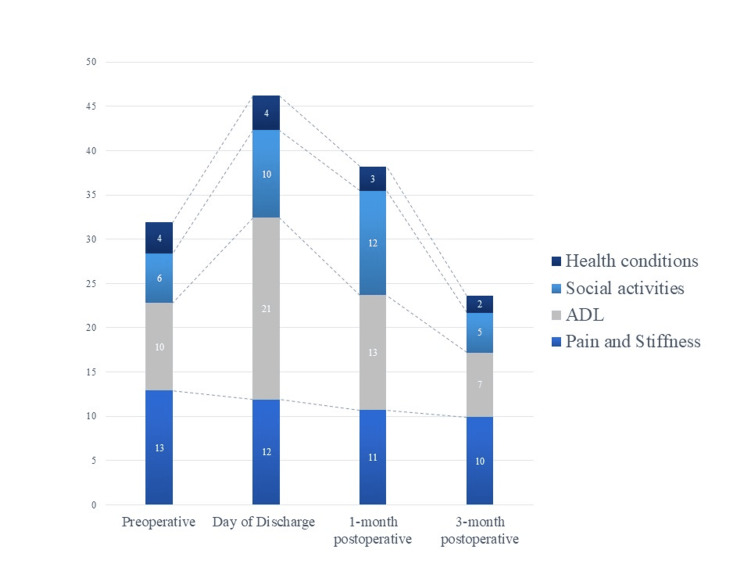
Breakdown of the results of the JKOM score JKOM: Japanese Knee Osteoarthritis Measure, ADL: Activities of daily living

**Figure 4:**
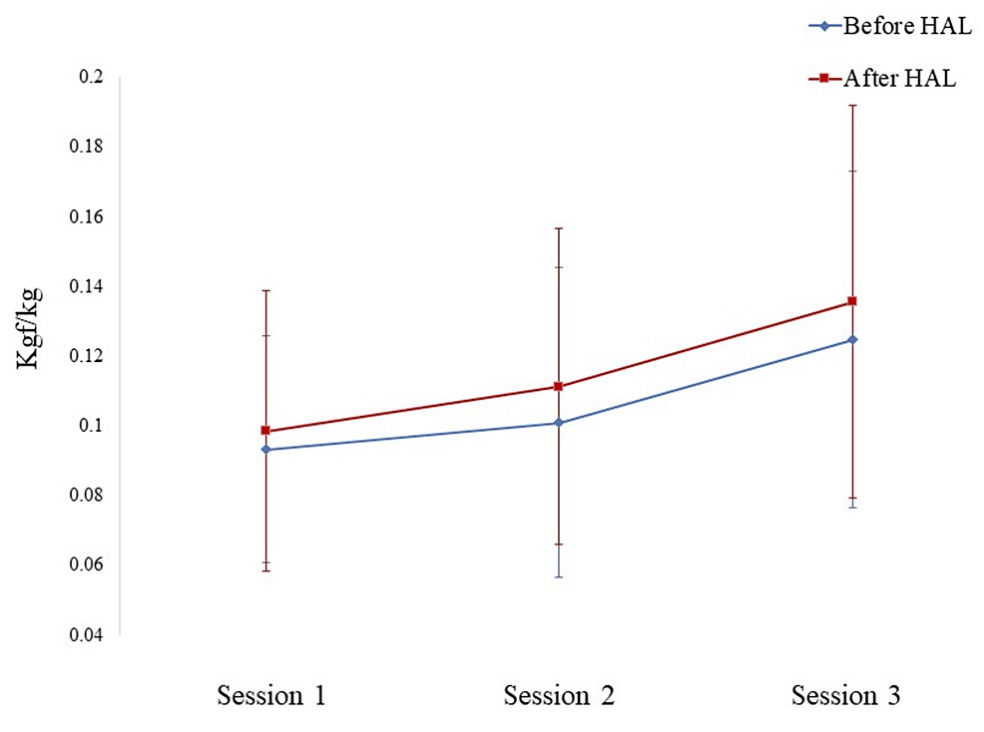
The HHD assessments both before and after each HAL training session HHD: Hand-held dynamometer, HAL: Hybrid assistive limb

**Figure 5:**
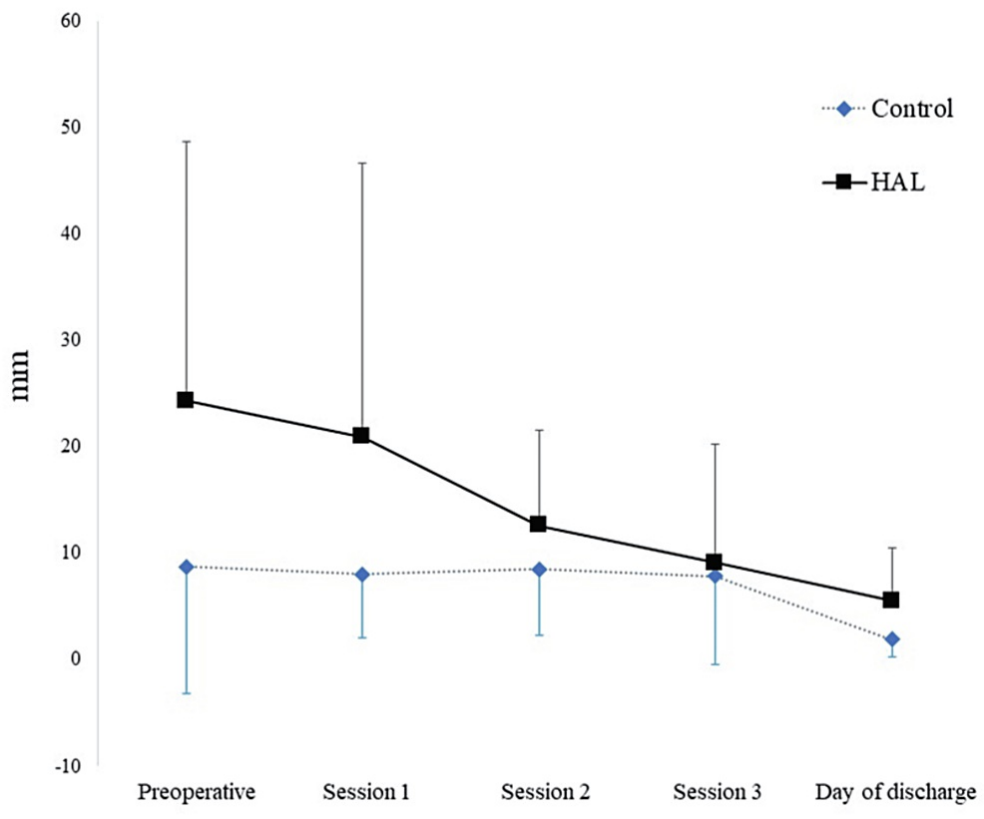
A two-way mixed analysis of variance of the VAS score (from before the surgery to the day of discharge) between the HAL and control groups VAS: Visual analog scale, HAL: Hybrid assistive limb

**Figure 6:**
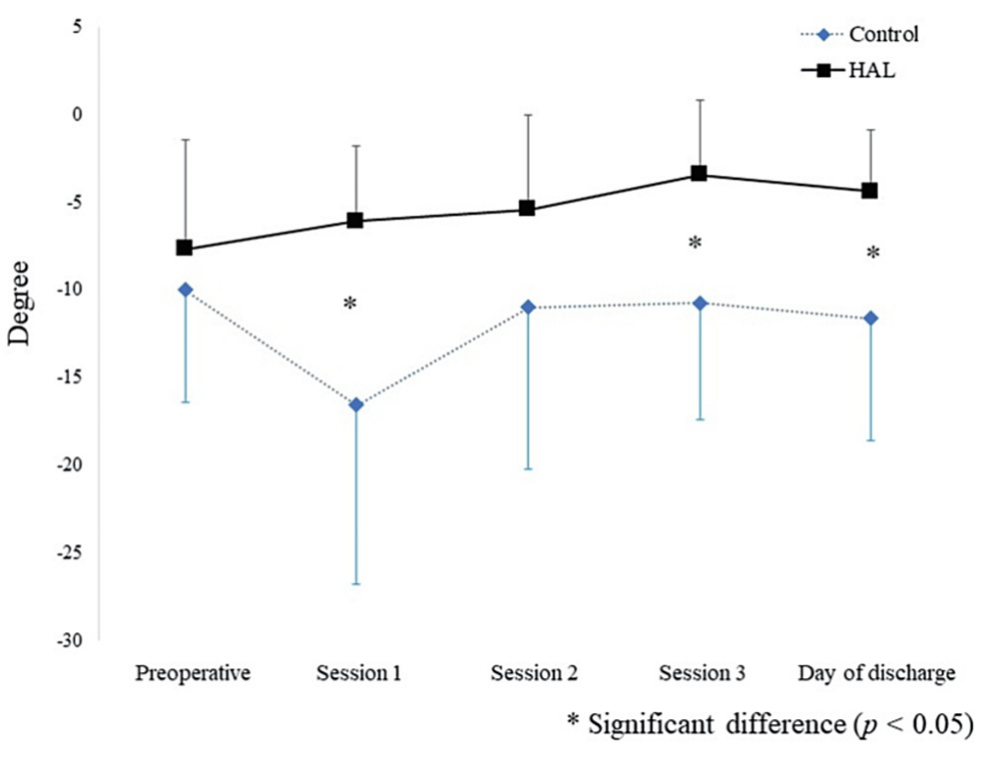
A two-way mixed analysis of variance and post hoc test of the knee extension angle (from before surgery to the day of discharge) between the HAL and control groups. HAL: Hybrid assistive limb

**Figure 7:**
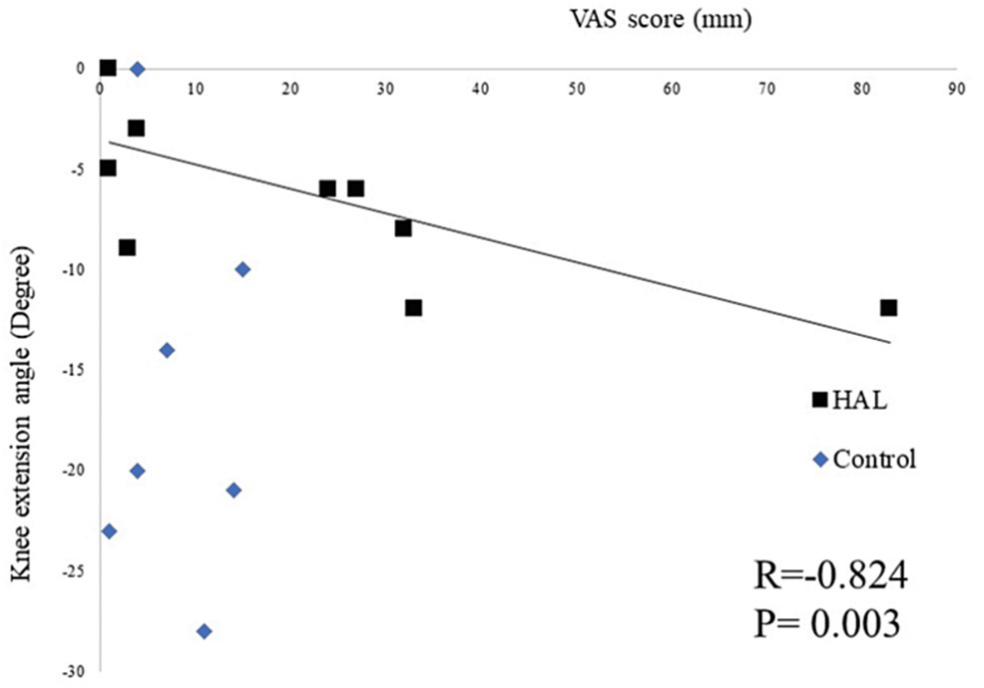
Correlations between the VAS score and knee extension angle in both HAL and control groups in the first session (p < 0.05) VAS: Visual analog scale, HAL: Hybrid assistive limb

**Figure 8:**
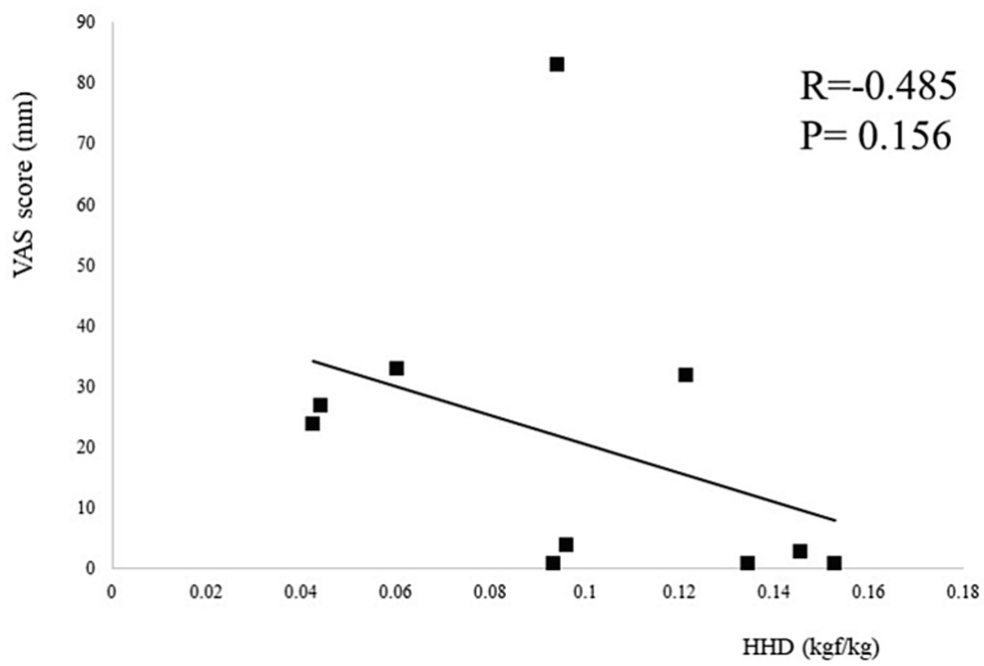
Correlation between the VAS score and the HHD assessments for the HAL group in the first session (p < 0.05) VAS: Visual analog scale, HHD: Hand-held dynamometer, HAL: Hybrid assistive limb

**Figure 9:**
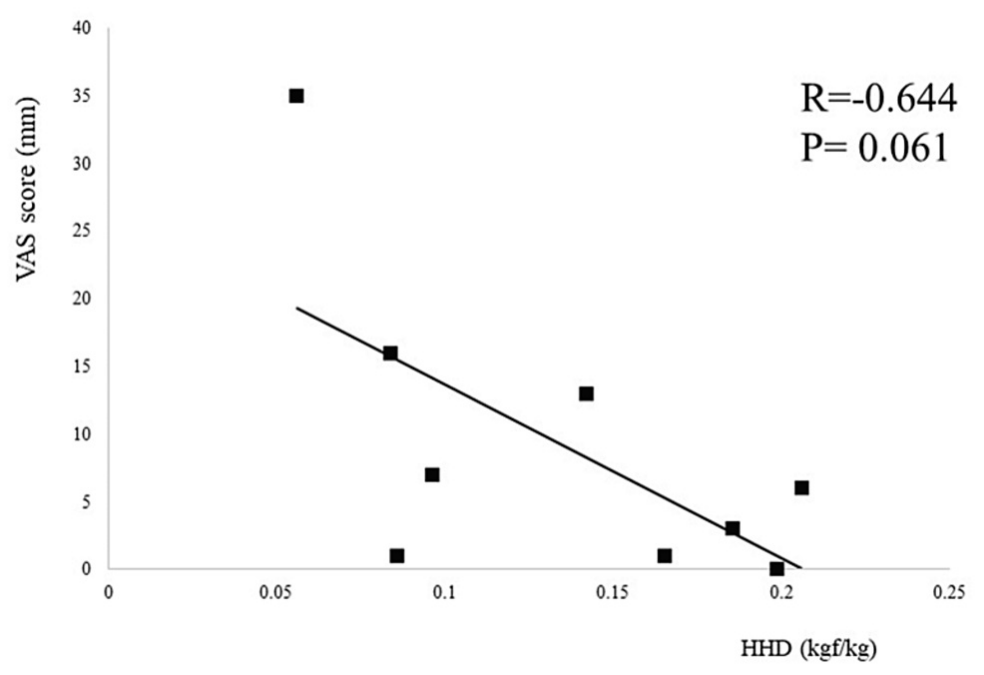
Correlation between the VAS score and the HHD assessments for the HAL group in the third session (p < 0.05) VAS: Visual analog scale, HHD: Hand-held dynamometer, HAL: Hybrid assistive limb

An itemization of the JOA scores and JKOM assessments is depicted in Figures 2-3. The HHD assessments were not significantly different pre- or post-training for any session (first session: p = 0.755, second session: p = 0.612, third session: p = 0.669) (Figure 4). The VAS score was not significantly different across all sessions (interaction effects: p = 0.286, ηp2 = 0.618, power = 0.266) (Figure 5). A two-way analysis of the knee extension angle assessments revealed significant differences (main effects: p = 0.000, ηp2 = 0.248, power = 0.997). Post hoc tests of the knee extension angle assessments exhibited a significant difference in the first session (p = 0.047), in the third session (p = 0.012), and on the day of discharge (p = 0.007), but not before the surgery or in the second session (before the surgery: p = 0.682, second session: p = 0.124) (Figure 6). The relationship between the VAS score and the knee extension angle in the HAL and control groups revealed significant negative correlations in the HAL group for the first session (r = -0.824, p = 0.003) (Figure 7). However, the second and third sessions revealed no significant correlations in either group. There was no significant relationship between the VAS score and the HHD assessment in the HAL group but low to moderate negative correlations for the first and third sessions (first session: r = -0.485, p = 0.156, third session: r = -0.644, p = 0.061) (Figures 8-9). The relationship between the knee extension angle and the HHD assessment in the HAL group was not significant.

## Discussion

We investigated the feasibility and safety of HAL-SJ training in the early postoperative period after OWHTO and determined whether the use of this device improves functional outcomes. This is the first original trial report demonstrating the applicability of HAL training for patients with OWHTO. We carefully assessed the suitability of HAL training for these patients by monitoring adverse events associated with HAL training and evaluating its impact on the clinical presentation. Remarkably, no adverse events were observed throughout the study, even during the early postoperative period. Furthermore, the assessment of clinical outcomes before and after OWHTO, which included parameters such as knee extension angle, the VAS score, HHD, the JOA score, and the JKOM at four distinct time points, demonstrated gradual improvement over time. In the comparison of clinical outcomes before and after OWHTO in the HAL group, these results showed gradual improvement compared to a previous study [21]. These findings support the feasibility and safety of HAL training in this trial.

In the HAL group, there was a slight increase in pain after discharge; however, statistical significance regarding this increase cannot be confirmed. Nevertheless, it is important to discuss the necessity of continuing HAL training even after discharge. In terms of postoperative clinical outcomes, a previous study suggested a strong correlation between postoperative clinical outcomes and patient satisfaction following a high tibial osteotomy. A previous study reported high patient expectations for ADL and pain relief after osteotomy around the knee [22]. Another study reported that knee injury and the osteoarthritis outcome score, pain, and ADL subscale scores were significant factors associated with patient satisfaction after OWHTO [21]. In our study, the JOA scores and JKOM assessments that include knee pain severity and self-reported physical function including ADL showed gradual improvement. Regarding the VAS score, there were no significant differences observed for any session between the HAL and control groups. However, in the HAL group, the VAS score gradually decreased over the course of the study. Notably, on the day of discharge, the VAS score was 5.5, a reduction compared to one and three months postoperatively. These results suggest that HAL training did not increase knee pain and could potentially be a safe tool, even in the early postoperative period during hospitalization.

Regarding the impact of HAL-SJ intervention, there was a significant improvement in knee extension angle during the first session, the third session, and on the day of discharge compared to the control group. Correlation analysis between the VAS score and knee extension angle during the first session demonstrated significant negative correlations only in the HAL group. This suggests that the HAL-SJ intervention may result in improved knee extension ROM with reduced knee pain in the early postoperative period. In a normal knee joint, the quadriceps muscle group contracts to generate an anterior shear force on the tibia relative to the femur. Simultaneously, the hamstring muscle group exhibits low-level activation to counteract excessive anterior translation of the tibia relative to the femur. This coordinated action between the quadriceps and hamstring muscles contributes to the stability of the knee joint during knee extension [23]. However, quadriceps weakness can lead to limitations in active extension.

Although TKA is effective, some patients may struggle to achieve full pre-surgical active ROM postoperatively. This limitation has been associated with progressive degenerative changes in knee arthritis. Soft tissue and bone damage during TKA can contribute to the development of active extension lag due to pain-induced inhibition of active muscle motor units [24]. In the context of this study, which focuses on OWHTO rather than TKA, there may be an impact on the impaired knee extensor mechanism and alterations in neuromuscular function due to knee arthritis, particularly in the early postoperative period. We hypothesized that training with the HAL-SJ device has the potential to impact the knee extensor mechanism. The HAL-SJ intervention may aid in optimizing the efficiency of muscle activity and ROM during movement as it possesses a bioelectric signal-balancing capability that can adjust the balance of detected flexion and extension signals through computer processing [25].

The HHD assessments were conducted both before and after each HAL training session, revealing slight improvement. However, this improvement was not statistically significant. When examining the relationship between the VAS score, HHD, and knee extension angle, negative correlations were observed for HHD and VAS scores. However, no correlation was observed between knee extension angle and HHD. These findings suggest that HAL training may contribute to reducing knee pain and supporting more efficient knee muscle contractions. Notably, the two-way analysis of knee extension range was significantly greater in the HAL group than in the controls. It is worth noting that the knee extension range might be more directly influenced by HAL, allowing the knee joint to move sufficiently and comfortably within its ROM [12].

Additionally, patients typically undergo a non-weight-bearing period following surgery. Per our institution's rehabilitation protocol, weight-bearing exercises are typically initiated on postoperative day 14. This delay in weight-bearing may contribute to alterations in electromechanical properties as a consequence of disuse, potentially resulting in a significant reduction in muscle strength [26]. Prior research has demonstrated a reduction in muscle fiber stiffness following a period of non-weight bearing in rats [27]. A decrease in the stiffness of the elastic components could lead to diminished efficiency in the transmission of contractile tension to the bones. Furthermore, human studies have reported changes in the tensile strength of the elastic components following disuse after orthopedic surgery [28]. Consequently, it is of paramount importance to emphasize targeting the knee extensor mechanism, particularly during the early postoperative period for individuals undergoing OWHTO.

Our study findings suggest that HAL-SJ training could play a role in decreasing knee pain, promoting more effective knee muscle contractions, and potentially preventing muscle atrophy. Initiating HAL-SJ training earlier post-surgery may be beneficial in preventing muscle atrophy. However, in this study, HAL training commenced on postoperative day 8 after assessing factors such as the patient's wound condition (presence of signs of infection, wound pain, wound swelling), and patient motivation. These parameters were monitored until the postoperative day 7. Preserving muscle strength and preventing muscle atrophy during non-weight-bearing phases may lead to more efficient weight-bearing improvements, enhancing activities such as walking and other activities of daily living in the mid-term postoperative period. Therefore, the utilization of HAL-SJ in patients who have undergone OWHTO appears to contribute to enhanced muscle activity efficiency by reducing knee pain and potentially improving knee extension angles in the early postoperative phase.

Limitations

In the context of the knee extensor mechanism, which may lead to changes such as the descent of the patella and an increase in patellofemoral joint pressure, it is crucial to highlight that this study lacks an outcome measure for evaluating compartmental changes using combined single-photon emission CT and conventional CT [29]. In this study, HAL training was implemented as an additional component to conventional rehabilitation. While one of the study's aims was to assess whether knee HAL-SJ training enhanced functional outcomes following OWHTO, discerning the recovery attributed to OWHTO itself from the potential impact of knee HAL training proved challenging. This study serves as a preliminary exploration and is constrained by its relatively small sample size. 

## Conclusions

Per our study, the HAL-SJ appears to be potentially safe in patients who undergo OWHTO. It contributes to enhanced muscle activity efficiency by reducing knee pain and improving knee extension angles in the early postoperative phase. Future investigations of the HAL-SJ should employ randomized trials and early assessments for comparison with a control group, with emphasis on distinct muscle characteristics through electromyographic analysis and specific pain assessments.
